# Supplementary material for: Volatilome and Essential Oil of Ulomoides dermestoides: A Broad-Spectrum Medical Insect
Source: Molecules. 2021 Oct 19;26(20):6311. doi: 10.3390/molecules26206311 (PMC8537694; doi:10.3390/molecules26206311)
Supplement: Supplementary file 1 [file molecules-26-06311-s001.zip › molecules-1410446-supplementary.pdf]

Volatilome and Essential Oil of *Ulomoides dermestoides*: A Broad-Spectrum Medical Insect.

Paulina J. Cázares-Samaniego [1], Claudia G. Castillo [1,*], Miguel A. Ramos-López [2] and Marco M. González-Chávez [3,*]

1 Coordinación para la Innovación de la Ciencia y la Tecnología (CIACYT), Facultad de Medicina, Universidad Autónoma de San Luis Potosí, Sierra Leona #550, Col. Lomas de San Luis, C.P.78210, San Luis Potosí, San Luis Potosí, México; xavet16@gmail.com (P.J.C.-S.), claudia.castillo@uaslp.mx (C.G.C.)
2 Facultad de Química, Universidad Autónoma de Querétaro, Cerro de las Campanas s/n, Col. Las Campanas, C.P. 76010, Santiago de Querétaro, Querétaro, México; miguel.angel.ramos@uaq.mx
3 Facultad de Ciencias Químicas, Centro de Investigación y Estudios de Posgrado, Universidad Autónoma de San Luis Potosí, Dr. Manuel Nava Martínez #6, Zona Universitaria, C.P.78210, San Luis Potosí, San Luis Potosí, México; gcmm@uaslp.mx
* Correspondence: M.M.G.-C. gcmm@uaslp.mx; Tel.: +52 4448262300 Ext. 2471; C.G.C. claudia.castillo@uaslp.mx Tel.: +52 4448262300 Ext. 8482

SUPPORTING INFORMATION

List of Contents

Figure S1. Gas chromatogram of the HS-SPME (CAR/PDMS fiber) at 1h of *U. dermestoides* under T1 treatment. 2

Figure S2. Gas chromatogram of the HS-SPME (PEG fiber) at 1h of *U. dermestoides* under T1 treatment.......... 2

Figure S3. Gas chromatogram of the HS- SPME (CAR/PDMS fiber) at 1h of *U. dermestoides* under T2 stimuli. 2

Figure S4. Gas chromatogram of the HS- SPME (PEG fiber) at 1h of *U. dermestoides* under T2 stimuli. 3

Figure S5. Gas chromatogram of *U. dermestoides* essential oil under T1 stimuli (EOT1)... 3

Figure S6. Gas chromatogram of *U. dermestoides* essential oil under T2 stimuli (EOT2)... 3

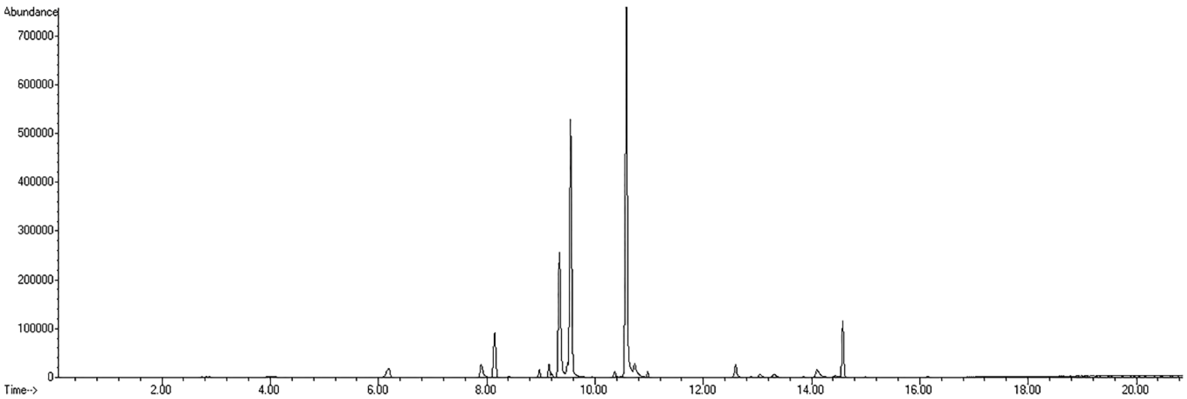

Figure S1. Gas chromatogram of the HS-SPME (CAR/PDMS fiber) at 1h of *U. dermestoides* under T1 treatment.

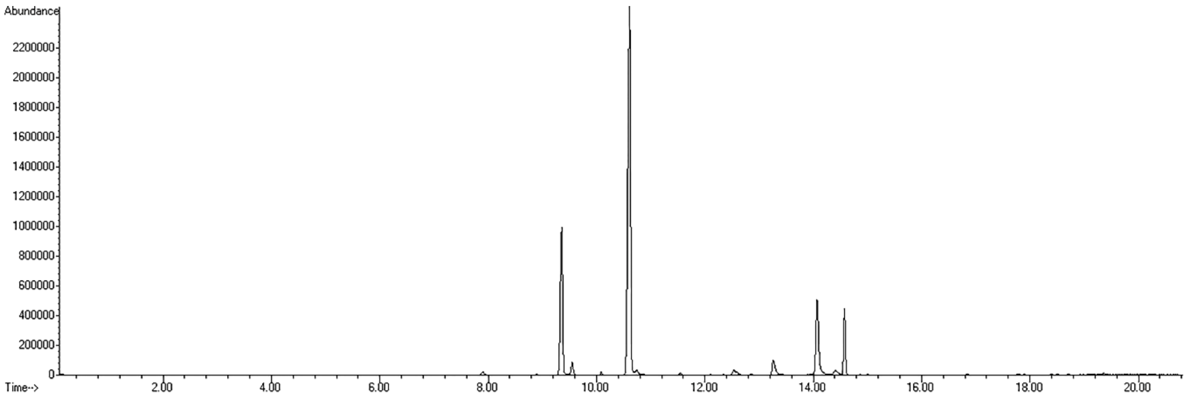

Figure S2. Gas chromatogram of the HS-SPME (PEG fiber) at 1h of *U. dermestoides* under T1 treatment.

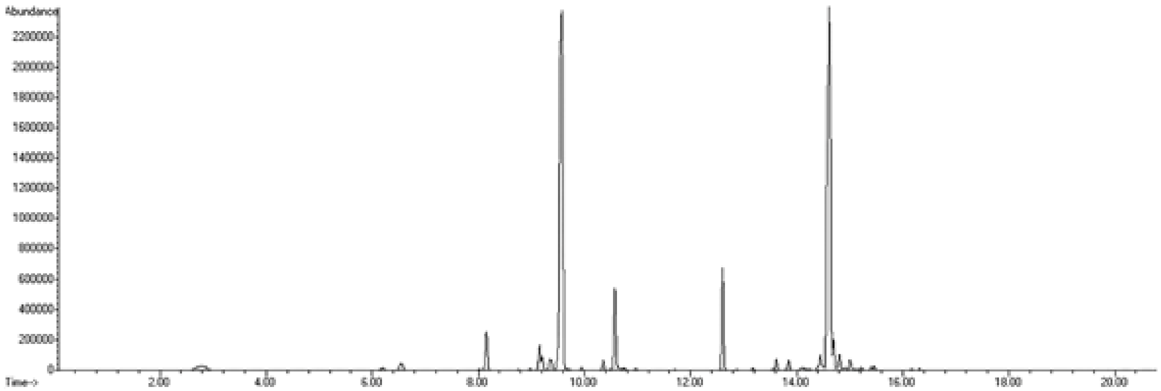

Figure S3. Gas chromatogram of the HS- SPME (CAR/PDMS fiber) at 1h of *U. dermestoides* under T2 stimuli.

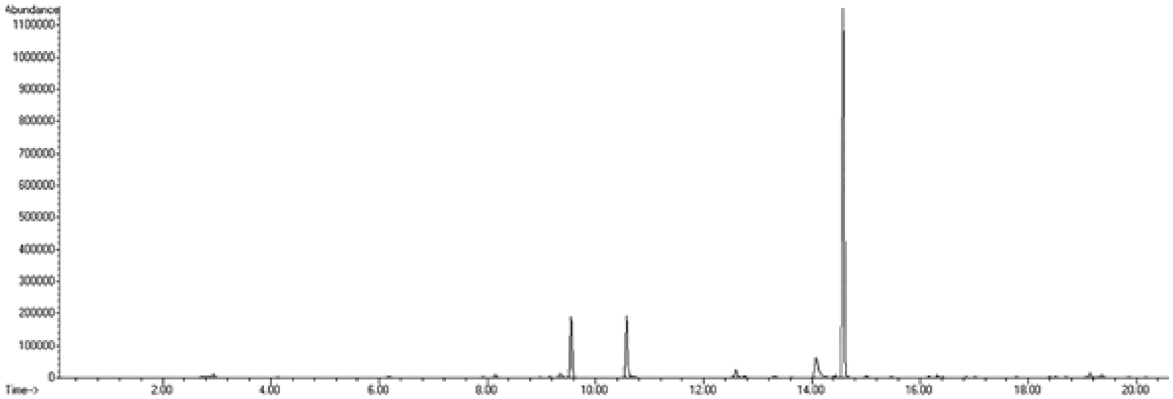

Figure S4. Gas chromatogram of the HS- SPME (PEG fiber) at 1h of *U. dermestoides* under T2 stimuli.

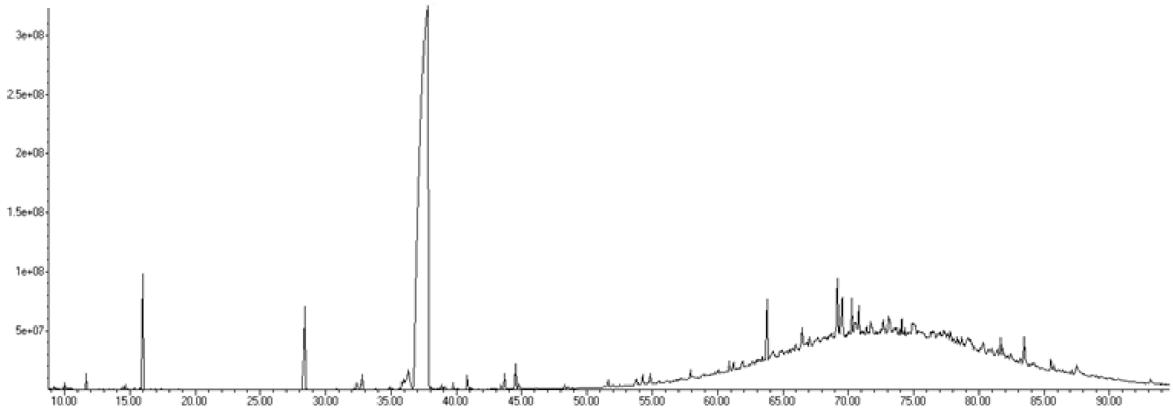

Figure S5. Gas chromatogram of *U. dermestoides* essential oil under T1 stimuli (EOT1).

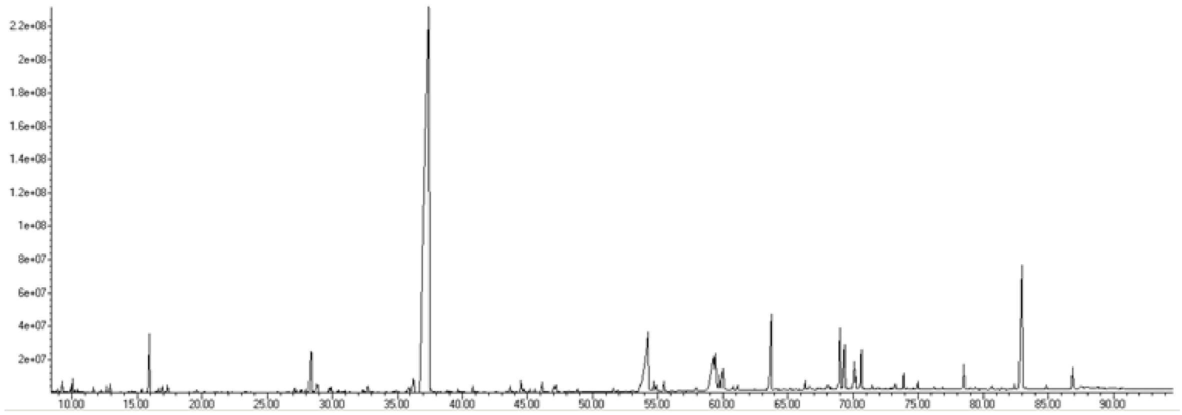

Figure S6. Gas chromatogram of *U. dermestoides* essential oil under T2 stimuli (EOT2).
